# Expression of Concern: MiRNA-574-3p inhibits cell progression by directly targeting CCND2 in colorectal cancer

**DOI:** 10.1042/BSR-20190976_EOC

**Published:** 2020-07-30

**Keywords:** apoptosis, colorectal cancer (CRC), Cyclin D2 (CCND2), microRNA-574-3p, migration, proliferation

The Editorial Office has been made aware of potential issues surrounding the scientific validity of this paper, hence has issued an expression of concern to notify readers whilst the Editorial Office investigates.

